# Tuneable red, green, and blue single-mode lasing in heterogeneously coupled organic spherical microcavities

**DOI:** 10.1038/s41377-020-00392-7

**Published:** 2020-08-28

**Authors:** Yuxiang Du, Chang-Ling Zou, Chunhuan Zhang, Kang Wang, Chan Qiao, Jiannian Yao, Yong Sheng Zhao

**Affiliations:** 1 CAS Key Laboratory of Photochemistry, Institute of Chemistry, Chinese Academy of Sciences, Beijing, 100190 China; 2 University of Chinese Academy of Sciences, Beijing, 100049 China; 3 Key Laboratory of Quantum Information, and Synergetic Innovation Center of Quantum Information and Quantum Physics, University of Science and Technology of China, Hefei, Anhui 230026 China

**Keywords:** Microresonators, Micro-optics

## Abstract

Tuneable microlasers that span the full visible spectrum, particularly red, green, and blue (RGB) colors, are of crucial importance for various optical devices. However, RGB microlasers usually operate in multimode because the mode selection strategy cannot be applied to the entire visible spectrum simultaneously, which has severely restricted their applications in on-chip optical processing and communication. Here, an approach for the generation of tuneable multicolor single-mode lasers in heterogeneously coupled microresonators composed of distinct spherical microcavities is proposed. With each microcavity serving as both a whispering-gallery-mode (WGM) resonator and a modulator for the other microcavities, a single-mode laser has been achieved. The colors of the single-mode lasers can be freely designed by changing the optical gain in coupled cavities owing to the flexibility of the organic materials. Benefiting from the excellent compatibility, distinct color-emissive microspheres can be integrated to form a heterogeneously coupled system, where tuneable RGB single-mode lasing is realized owing to the capability for optical coupling between multiple resonators. Our findings provide a comprehensive understanding of the lasing modulation that might lead to innovation in structure designs for photonic integration.

## Lasers: Generating all colors in a better mode

A system for improved control over laser light emission generates any frequency in the visible spectrum, creating what is termed a red, green and blue (RGB) laser, in the advantageous form known as ‘single-mode’ lasing. Most RGB lasers deliver ‘multimode’ signals, in which the light is distributed among several wavelengths around the central most intense and desired wavelength. The preferable single-mode RGB lasing has been developed by researchers in China, led by Yong Sheng Zhao at the Institute of Chemistry of the Chinese Academy of Sciences in Beijing. It uses closely coupled organic (carbon compound-based) microcavities as the regions in which the light is generated and amplified, delivering single frequencies of tunable laser light. This innovation will open new possibilities for using RGB lasing in optical processing and communications systems.

## Introduction

Tuneable microlasers that span the full visible spectrum are essential building blocks for lighting technology, full color laser display, and sensing^1–4^. Due to the inhomogeneous gain saturation introduced by spatial hole burning, most wavelength-tuneable microlasers are subject to operation in multimode, which will lead to temporal fluctuations and false signaling^5–8^. To date, several mode manipulation strategies have been proposed to realize single-mode lasing in well-designed structures by using distributed feedback gratings^9^, spatially varying optical pumps^10^, and parity-time symmetry breaking^5,6^. However, most of the strategies allow the achievement of single-mode lasing in only one gain region due to the intrinsic difficulties in simultaneously fabricating distinct materials. As a key requirement of digitized communications and signal processing, the generation of tuneable single-mode microlasers capable of emitting over the full visible spectrum, particularly the red, green, and blue (RGB) color regions, remains a great challenge, which has been a major obstacle limiting their practical applications^3^.

Until now, RGB microlasers were achieved mainly by integrating different gain media into single photonic devices^11–15^, which usually suffer from operating in multimode. Expanding the free spectral range (FSR) by reducing the cavity size is effective for multicolor single-mode microlasers^16^, which can be applied to different wavelengths simultaneously but may increase the threshold^17^. Coupled cavities, with one cavity applied as a modulator of the other, could enable the expansion of the FSR while avoiding an obvious increase in threshold and have been proven to be an ideal platform to achieve single-mode lasing^18–21^. By incorporating different optical gains into the respective resonators, dual-color single-mode lasers have been realized in axially coupled cavity systems^8^. Unfortunately, this axial coupling strategy cannot simultaneously act in RGB wavebands due to the direct coupling along the cavity axis between two Fabry–Pérot cavities^22^. Whispering-gallery-mode (WGM) resonators, permitting guided light coupling in the surrounding medium^23–25^, can serve as photonic units to construct coupled systems with a large number of interacting optical microcavities^18^, which are potential candidates to achieve tuneable RGB single-mode microlasers. However, realizing heterogeneously coupled microstructures with conventional semiconductors is limited by the difficulties in the fabrication and manipulation of regularly shaped RGB-emissive resonators as a result of the poor compatibility. Organic materials, benefiting from excellent flexibility, possess the ability to self-assemble into well-defined WGM spherical cavities^26,27^. Moreover, the compatibility of organic materials enables the incorporation of various gain media with lasing wavelengths across the entire visible spectrum^28,29^, making these materials promising for constructing heterogeneously coupled microstructures for tuneable RGB single-mode microlasers.

Here, we demonstrate tuneable RGB single-mode lasing in heterogeneously coupled cavities constructed with three spherical microcavities incorporating distinct gain media. Microcavities with perfect circular boundaries and smooth surfaces were fabricated through liquid-phase assembly and could be applied as WGM resonators. Owing to the outstanding flexibility, RGB lasing was obtained by doping different dyes into the respective microcavities. Distinct color-emissive spherical cavities were integrated to construct an optimized heterogeneously coupled system through a micromanipulation technique. With the modulation of the coupled structures, a dual-color single-mode laser was realized from the heterogeneously coupled system. Because of the excellent compatibility of organic materials, a heterogeneously coupled system composed of RGB-emissive microcavities was fabricated. Benefiting from the capability for optical coupling between multiple resonators, tuneable RGB single-mode microlasers were realized. The results not only provide new insights into the achievement of microlasers spanning the full visible spectrum with high spectral purity but also support innovation of photonic units in optoelectronic integrated systems, such as undefined laser displays and integrated optical circuits.

## Results

**Fig. 1:**
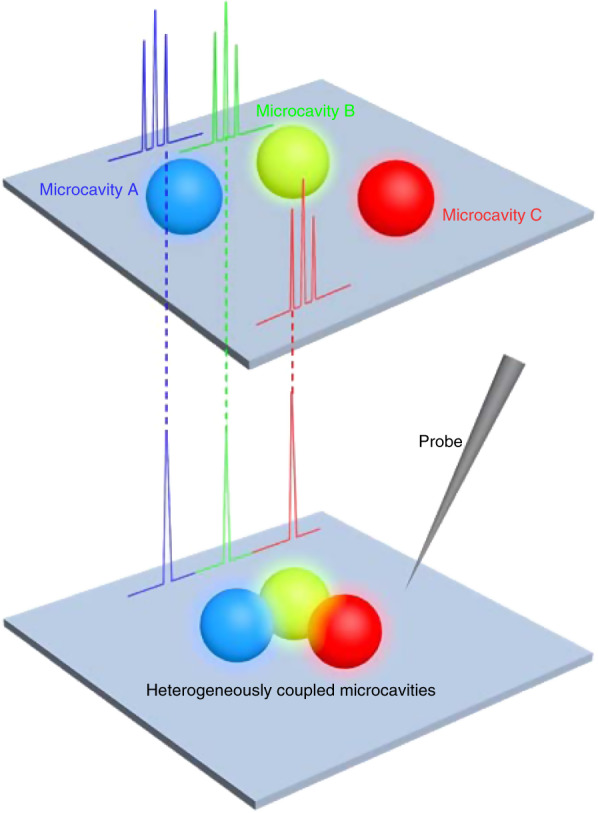
Mode selection concept in the heterogeneously coupled resonant cavities. Sketch of the multimode lasing from isolated microspheres (top), and RGB single-mode lasing from heterogeneously coupled microcavities (bottom), where each microsphere functions as not only the laser source but also the mode modulator for the other resonators.

The design principle for the realization of an RGB single-mode laser is schematically presented in Fig. 1. Isolated dye-doped microspheres can serve as WGM resonant cavities, with which multimode RGB microlasers were achieved through doping of corresponding laser dyes. Here, a strategy was proposed to achieve an RGB single-mode microlaser by building a heterogeneously coupled system composed of three interacting microresonators. RGB-emissive spherical resonators were integrated with a micromanipulator. The WGM resonators were arranged side by side due to the strong coupling of the optical field distributed along the cavity interface^18,22^. The dye-doped microspheres therein steadily deliver multimode lasing, while coupled microcavities act as filters of the resonance modes. With each spherical cavity serving as a laser source and a modulator simultaneously, RGB single-mode laser output would be achieved in heterogeneously coupled microcavities. Moreover, a tuneable RGB single-mode laser might be obtained by varying the manner of the optical pumping.

**Fig. 2:**
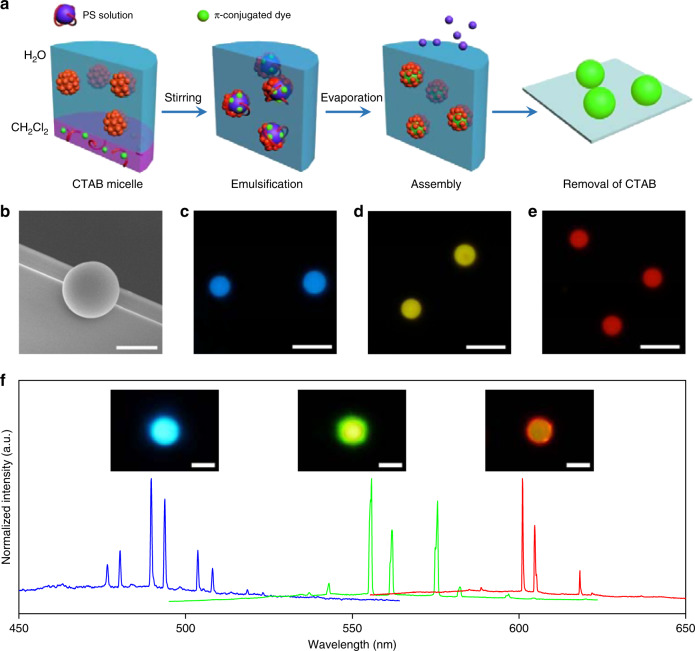
Preparation of the WGM resonators and RGB microlasers. **a** Schematic diagram of the microsphere fabrication process. **b** SEM image of the PS microsphere. Scale bar is 5 μm. **c**–**e** PL microscopy images of the microspheres doped with C153, CNDPASDB, and DCM, respectively, under UV excitation. Scale bars are 10 μm. **f** Multimode lasing spectra from the microspheres doped with C153, CNDPASDB, and DCM, excited with a pulsed laser (400 nm). Insets: corresponding PL images of the dye-doped microspheres above the lasing threshold. Scale bars are 5 μm.

The fabrication of organic spherical microcavities incorporating laser dyes is illustrated in Fig. 2a. Polystyrene (PS), due to its flexibility and processability, was selected as the matrix material to fabricate the microspheres. Well-mixed conjugated dye/PS/dichloromethane (CH_2_Cl_2_) solution was added into a cetyltrimethylammonium bromide (CTAB) aqueous solution, which formed an oil-in-water emulsion. After vigorous stirring, the mixed CH_2_Cl_2_ solution was encapsulated into the hydrophobic interior of CTAB. With the evaporation of CH_2_Cl_2_, spherical droplets consisting of PS molecules aggregated into microspheres with dye molecules dispersed inside. After the removal of CTAB, dye-doped spherical microcavities with uniform size were acquired.

The spherical geometry of the self-assembled microcavities was confirmed by top-view (Fig. 2b) and side-view (Supplementary Fig. 1) scanning electron microscopy (SEM) images. With perfect circular boundaries and ultrasmooth surfaces, the self-assembled microspheres minimize undesirable optical scattering, which is favorable for WGM resonance^30^. Based on the formation mechanism mentioned above, the diameter of self-assembled microcavities is directly proportional to the size of the micelles, which depends on the interfacial tension between water and the CH_2_Cl_2_ solution. The interfacial tension positively increases with the amount of PS, generating larger micelles with smaller specific surface areas to reduce the interfacial energy. Accordingly, the diameter of the self-assembled microsphere was finely tuned from 3 to 20 μm by changing the concentration of PS molecules, which is essential for constructing an optimized heterogeneously coupled cavity system (Supplementary Figs. 2 and 3). Due to the π–π interactions between the phenyl groups of PS and π-conjugated laser dyes, the self-assembled PS microspheres can be doped with various conjugated dyes to provide optical gains at different wavebands^31^. Three laser dyes were selected to serve as gain media: coumarin-153 (C153), 1,4-bis(α-cyano-4-diphenylaminostyryl)-2,5-diphenylbenzene (CNDPASDB; Supplementary Fig. 4), and 4-(dicyanomethylene)-2-methyl-6-(4-dimethylaminostyryl)-4H-pyran (DCM), which have photoluminescence (PL) emissions in blue, green, and red wavebands, respectively (Supplementary Fig. 5).

The self-assembled microcavities doped with C153, CNDPASDB, and DCM emitted uniform blue, green, and red fluorescence, respectively, under ultraviolet (UV) excitation (Fig. 2c–e). The introduction of various conjugated dyes caused very little surface damage to the microspheres due to the superior compatibility of organic materials (Supplementary Fig. 6), making them ideal candidates for lasing action. When each dye-doped microsphere was pumped with a pulsed laser beam (400 nm, ~200 fs) in a homebuilt microphotoluminescence system (Supplementary Fig. 7), multimode lasing action was observed (Fig. 2f). The linewidth of the individual lasing mode was ~0.5 nm. The quality factor of such spherical microcavities was determined to be ~1600, indicating the low optical loss of the microcavities^32^. The PL images of the dye-doped microspheres recorded above the thresholds exhibited bright ring-shaped patterns at the boundary (Fig. 2f, inset), which is a typical characteristic of WGM resonances^33^. Further investigation of the FSR showed that the mode spacing is inversely proportional to the diameter of the microspheres, verifying the WGM resonance (Supplementary Fig. 8)^34^.

The WGM oscillation in the spherical microcavity would result in the optical field being confined along the cavity interface, which enables strong coupling in a side-by-side coupled structure^18^. Accordingly, a heterogeneously coupled WGM resonator composed of a C153-doped microsphere and a CNDPASDB-doped microsphere was designed to modulate the laser output from the spherical microcavities. Here, a micromanipulation technique (Supplementary Fig. 9) was applied to controllably fabricate these heterogeneously coupled microcavity systems with desired structural parameters, including the diameter of the coupled microcavity (Supplementary Fig. 10) and intercavity gap distance (Supplementary Fig. 11), which provides a reliable means of precisely constructing the heterogeneously coupled microstructure.

**Fig. 3:**
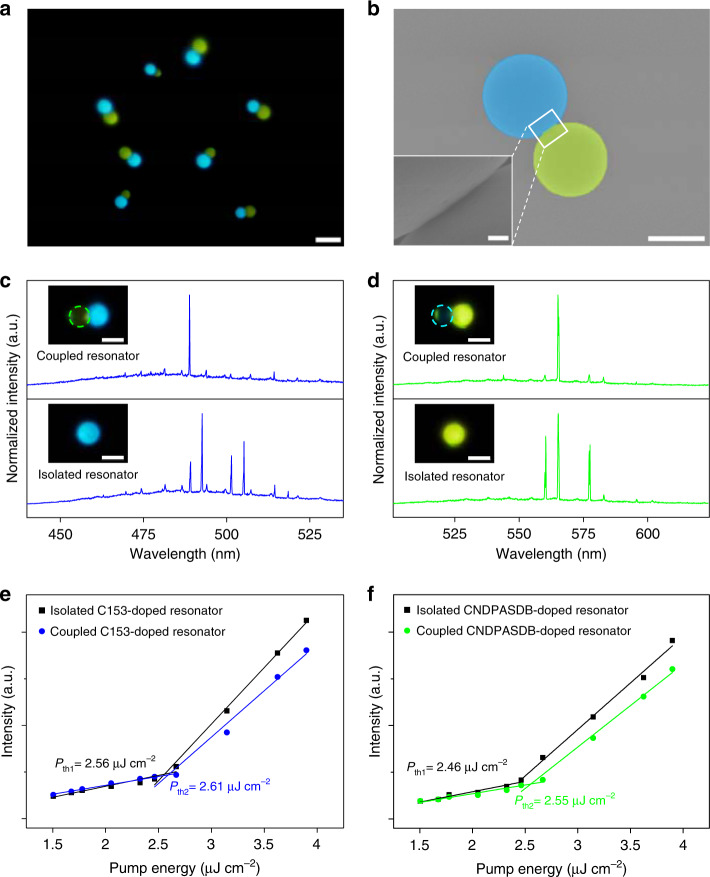
Realization of single-mode lasing in heterogeneously coupled microspheres. **a** PL image of the heterogeneously coupled microspheres under UV excitation. Scale bar is 10 μm. **b** False-colored SEM image of the coupled WGM resonators. Scale bar is 5 μm. Inset: magnified view of the gap region. Scale bar is 100 nm. Transition of the lasing spectra of the C153-doped microsphere (**c**) and CNDPASDB-doped microsphere (**d**) from multimode to single mode when coupled with distinct microsphere. Insets: corresponding PL images of the individual microsphere and the coupled microsphere under laser excitation. Scale bars are 5 μm. **e**–**f** Pump power-dependent PL intensities of the isolated and heterogeneously coupled resonators shown in (**c**) and (**d**).

The morphology of the as-prepared heterogeneously coupled resonators was demonstrated in PL (Fig. 3a) and SEM (Fig. 3b) images. Under UV excitation, the heterogeneously coupled microcavities maintain uniform emission without evident scattering points. The result manifests that the micromanipulation technique introduced little surface damage to the microspheres, which is supported by the SEM image. Hence, the heterogeneously coupled resonators constructed with the micromanipulation process preserve the optical properties of the individual WGM microcavities. The distance between microspheres in each heterogeneously coupled system was controlled to the nanometer scale (inset of Fig. 3b), enabling effective optical interaction between the resonators (Supplementary Fig. 12)^18^.

The lasing action in the heterogeneously coupled system was investigated by comparing the lasing spectra of identical WGM resonators with and without the coupling of distinct microspheres. The lasing spectrum of an isolated C153-doped microsphere exhibited a series of sharp peaks (Fig. 3c, top). In contrast, when the same microsphere was heterogeneously coupled with a distinct microcavity, one of the lasing modes in the isolated resonator was selectively oscillated in the coupled resonator, enabling blue single-mode lasing (Fig. 3c, bottom). Such a phenomenon could also be found in the green-emissive microcavity, indicating that mode selection in the heterogeneously coupled system could be achieved in other color regions. As shown in Fig. 3d, one of the multiple laser modes in an isolated CNDPASDB-doped microsphere was selected when coupled with a C153-doped microsphere, and green single-mode lasing was generated. The single-mode microlasers can produce steady output when the gap distance between coupled microcavities is varied from 0 to 250 nm (Supplementary Fig. 13), indicating that the mode selection effect has a low requirement on the gap distance. The thresholds of the microspheres doped with C153 and CNDPASDB in the coupled system were ~2.61 and 2.55 μJ cm^−2^, respectively, slightly higher than those of the isolated resonators (~2.56 and 2.46 μJ cm^−2^) (Fig. 3e, f). The slight increase in the lasing threshold can be ascribed to the radiation loss introduced by the coupled structure^35^.

**Fig. 4:**
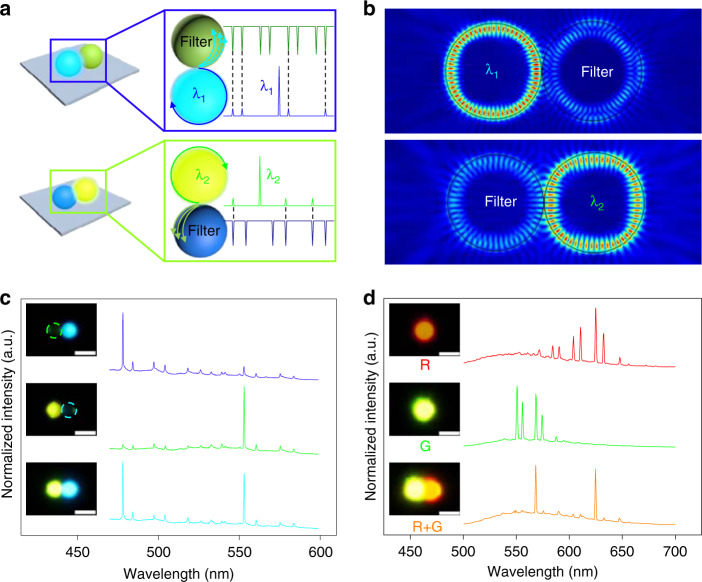
Mechanism of mode selection in the heterogeneously coupled system. **a** Schematic illustration of the mode selection mechanism. **b** Numerically simulated electric field distributions of the lasing modes in heterogeneously coupled resonators, which manifest the mode modulation in the heterogeneously coupled system. **c** Lasing emission spectra of the heterogeneously coupled cavities under laser excitation at different positions. Scale bars are 5 μm. **d** Transition of the normalized lasing spectra of the typical DCM-doped microsphere and CNDPASDB-doped microsphere from multimode to single mode when they are coupled with each other. Inset: corresponding PL images of the individual CNDPASDB-doped microsphere and DCM-doped microsphere and the coupled microsphere pairs under laser excitation. Scale bars are 5 μm.

The mechanism behind the generation of a single-mode laser in the heterogeneously coupled microcavities is shown in Fig. 4a. In the coupled system, the generated light propagates around the circumference of the lasing cavity, which makes the guided waves accessible for coupling to the external cavity. When the emitted light is coupled to the external WGM resonator, a series of sharp dips are observed in the transmission spectrum^18^, which can be attributed to the transmission resonance of the external cavity. When the transmission dips overlap the resonant frequencies of the lasing cavity, the optical power near these resonant frequencies transfer to the external cavity, resulting in a filtering effect^23^. By contrast, the optical power near the least overlapped resonant frequency of the lasing cavity has the lowest leakage into the passive cavity. Because of the lowest radiation loss introduced by the filter cavity, single-mode lasing at this resonant frequency will be achieved in the lasing cavity (Fig. 4a, top)^35,36^. Thus, the passive cavity serves as a filter of the lasing modes in the active cavity, which leads to a mode selection effect^37^. Such a mode selection strategy can also act on other wavebands because transmission dips of the filter cavity exist in other gain regions. When the green-emissive microsphere serves as the lasing cavity, single-mode lasing in the green waveband can be realized with the coupling of a filter cavity (Fig. 4a, bottom).

The mode selection mechanism mentioned above provides us with a strategy to achieve single-mode laser emission in different wavebands, which is supported by the simulated electric field distributions. As shown in Fig. 4b (top), the lasing mode (*λ*_1_ = 486.3 nm) is well confined in the left WGM cavity because of the low transmission loss introduced by the filter cavity at *λ*_1_, resulting in blue single-mode lasing action^35,37^. In the same coupled system, when the right resonator serves as the lasing cavity (Fig. 4b, bottom), another lasing mode (*λ*_2_ = 568.1 nm) dominates the right WGM cavity, and single-mode lasing in another gain region can be realized. With the two WGM resonators in the heterogeneously coupled system providing different optical gains, both of them can be applied as laser cavities. Modulated by the right WGM cavity, single-mode lasing can be achieved in the left microcavity, and vice versa. This result indicates that such a mode selection mechanism can act on the distinct gain regions in an identical heterogeneously coupled system, which has great potential for generating multicolor single-mode lasing.

This predicted result was confirmed by experimental measurements. In a heterogeneously coupled system constructed with a C153-doped microsphere and a CNDPASDB-doped microsphere, blue and green single-mode lasers, respectively, can be emitted from the two resonators (Fig. 4c, top and middle). A blue single-mode laser is obtained when the CNDPASDB-doped microsphere acts as a modulator for the C153-doped lasing cavity, whereas the C153-doped microcavity serves as a modulator for the generation of a green single-mode laser. These single-mode lasing behaviours indicate mutual mode selection, which would enable multicolor single-mode lasing when the heterogeneously coupled resonators serve as lasing cavities and mode filters simultaneously. As shown in Fig. 4c (bottom), a dual-color single-mode laser was achieved by pumping the entire heterogeneously coupled system. The pump power-dependent PL spectra of the dual-color single-mode lasing and plots of the pump power-dependent full-width at half-maximum are shown in Supplementary Fig. 14, verifying the multicolor single-mode lasing in the heterogeneously coupled resonators.

The colors of dual-wavelength single-mode lasers might be freely designed by varying the gain medium in the lasing cavities. As shown in Fig. 4d (top and middle), a DCM-doped microsphere and a CNDPASDB-doped microsphere were selected as lasing cavities in another coupled system because of their ability to realize red and green microlasers, respectively (Supplementary Fig. 15). When these two microcavities were heterogeneously coupled with each other, red and green single-mode lasing was realized in the coupled microcavities (Fig. 4d, bottom). This result shows that by building a coupled system with distinct microcavities, a single-mode laser covering all visible colors can be achieved based on the mode selection mechanism. Meanwhile, benefiting from the isotropic emission of the WGM resonator, the spherical microcavity could permit optical coupling with multiple microcavities simultaneously, which enables us to construct a coupled system composed of more resonators^38^. Such heterogeneously coupled systems may provide a general strategy for the generation of single-mode lasers covering a wider wavelength region.

**Fig. 5:**
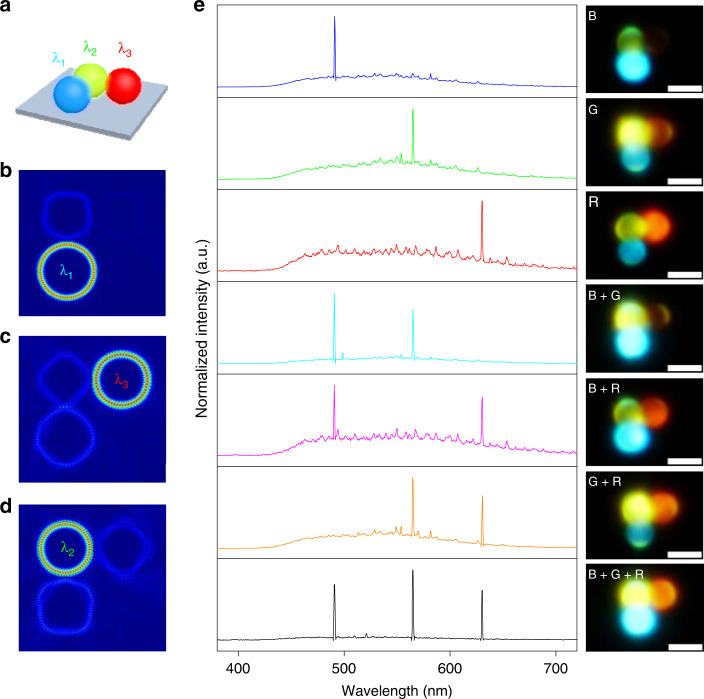
Tunable RGB single-mode lasing from heterogeneously coupled WGM resonant cavities. **a** Schematic illustration of the heterogeneously coupled system constructed with red-, green- and blue-emissive microcavities. **b**–**d** Numerically simulated electric field distributions of the lasing modes in coupled system containing three resonators, demonstrating the mode selection in three-component coupled system. **e** Normalized single-mode lasing emission spectra and corresponding PL images when different positions of the heterogeneously coupled system were pumped above their thresholds. From top to bottom: the blue (B), green (G), red (R), blue and green (B + G), blue and red (B + R), green and red (G + R), and blue, green, and red (B + G + R) emissive spherical microcavities. Scale bars are 5 μm.

The outstanding compatibility and isotropic emission of the spherical microcavities permitted us to design a three-component coupled system capable of simultaneously achieving RGB microlasers with optical coupling between them. The red-, green-, and blue-emissive microcavities were arranged into an angular-shaped chain structure, which not only enabled the interaction between the microcavities but also allowed us to simultaneously pump any two of the resonators, as shown in Fig. 5a. In such a heterogeneously coupled system, RGB single-mode lasers might be obtained in distinct microcavities, which is supported by the numerically simulated electric field distributions of the lasing modes (Fig. 5b–d). The green-emissive microcavity serves as a filter for the blue- and red-emissive microcavities, which leads to blue (*λ*_1_ = 483.6 nm) and red (*λ*_3_ = 610.2 nm) single-mode lasing. Meanwhile, the green-emissive microcavity is synchronously modulated by the other two resonators, and the lasing mode (*λ*_2_ = 554.3 nm) is mainly located inside the WGM resonator, which results in green single-mode lasing.

Indeed, tuneable RGB single-mode lasing was experimentally observed in such a three-component heterogeneously coupled system. The coupled cavity is composed of a DCM-doped microsphere, a CNDPASDB-doped microsphere, and a C153-doped microsphere. As shown in Fig. 5e, when each individual microsphere cavity was pumped above the threshold, single-mode lasing was achieved at the corresponding wavelength. When two of the coupled microcavities were pumped above their thresholds, any light combination comprising two of the RGB single-mode lasers could be generated. Tuneable multicolor single-mode lasers (B + G, G + R, and B + R) were obtained by adjusting the manner of the optical pumping, and an RGB single-mode laser was achieved when all three microspheres were integrally pumped. Such tuneable RGB single-mode laser output from the coupled system is desirable for full utilization of the advantages of RGB microlasers, which would greatly contribute to ultracompact photonic devices^39–41^.

## Discussion

In summary, we have developed a general strategy for the generation of tuneable multicolor single-mode lasers in heterogeneously coupled organic spherical microcavities. In such a heterogeneously coupled system, each individual microsphere serves as not only a laser source but also a modulator for the other resonators, which enables single-mode lasing from individual microcavities. The wavelength of a single-mode laser can be freely designed by changing the optical gain in coupled cavities due to the material compatibility of organic microspheres. Based on the mode selection mechanism in the coupled resonator, a three-component coupled system was designed, and tuneable RGB single-mode lasers were realized. These results reshape the understanding of lasing modulation in heterogeneously coupled systems and promote the development of photonic units in optoelectronic integrated systems.

## Materials and methods

### Materials

#### Matrix materials

Polystyrene (M.W. 250,000), which was purchased from J&K Scientific Ltd. (Beijing, China), was selected as the matrix material to create high-quality spherical resonators due to its outstanding flexibility.

#### Laser dyes

Coumarin 153 (C153, 97%), 1,4-bis(α-cyano-4-diphenylaminostyryl)-2,5-diphenylbenzene (CNDPASDB), and 4-(dicyanomethylene)-2-methyl-6-(4-dimethylaminostyryl)-4H-pyran (DCM, 99%), which exhibit photoluminescence at blue, green, and red wavebands, respectively, were selected as gain media. C153 was purchased from J&K Scientific Ltd. (Beijing, China). DCM was purchased from Acros Organics (Beijing, China). CNDPASDB was synthesized with Knoevenagel condensation reactions (Supplementary Fig. 3).

#### Starting materials of CNDPASDB

Potassium tert-butoxide, tetra-butyl ammonium hydroxide, 1,4-dibromo-2,5-dimethylbenzene, and 4-(diphenylamino)benzaldehyde were purchased from Aldrich. 2,5-Dibromobenzene-1,4-dicarbaldehyde was purchased from InnoChem Science & Technology (Beijing, China).

#### Other materials

CTAB purchased from InnoChem was used to form spherical micelles in aqueous solution, which is a key factor for controlling the diameter of self-assembled microcavities.

### Preparation

#### Preparation of dye-doped microspheres

Dye-doped PS spherical microcavities were fabricated through a liquid-phase assembly method. In a typical preparation, 110 μL well-mixed dye/PS/dichloromethane (CH_2_Cl_2_) solution was added into 1 mL CTAB aqueous solution (2 mmol). After vigorous stirring, dye-doped PS microspheres were obtained in the colloid solutions. Later, the CTAB was removed through filtration and washing. The spherical microcavities were redispersed in aqueous solution and then used to prepare samples for further characterization by drop-casting. The diameters of the as-prepared spherical microcavities can be well tuned from 3 to 20 μm by increasing the concentration of PS from 10 to 50 mg mL^−1^. The laser dyes were added to the polymer solution at a concentration of ~1 wt.%.

## Supplementary information

Supplementary Information

## Data Availability

The data that support the findings of this study are available from the corresponding author upon reasonable request.

## Code availability

The finite-difference time-domain source code can be accessed from http://www.fdtdxx.com.
