# Supplementary material for: Tuneable red, green, and blue single-mode lasing in heterogeneously coupled organic spherical microcavities
Source: Light Sci Appl. 2020 Aug 28;9:151. doi: 10.1038/s41377-020-00392-7 (PMC7455725; doi:10.1038/s41377-020-00392-7)
Supplement: Supplementary file 1 — Supplementary Information [file 41377_2020_392_MOESM1_ESM.pdf]

Supplementary Information

Tunable Red, Green and Blue Single-mode Lasing in Heterogeneously Coupled Organic Spherical Microcavities

Yuxiang Du[1,3], Chang-Ling Zou[2], Chunhuan Zhang[1,3], Kang Wang[1,3], Chan Qiao[1,3], Jiannian Yao[1,3], Yong Sheng Zhao[1,3*]

[1]CAS Key Laboratory of Photochemistry, Institute of Chemistry, Chinese Academy of Sciences, Beijing 100190, China

[2] Key Laboratory of Quantum Information, and Synergetic Innovation Center of Quantum Information and Quantum Physics, University of Science and Technology of China, Hefei, Anhui 230026, China

[3] University of Chinese Academy of Sciences, Beijing 100049, China

*e-mail: yszhao@iccas.ac.cn

Contents

I. Characterization

II. Morphological characterization of spherical microcavities

Supplementary Figure 1 | Top- and side-view SEM images of the PS microspheres.

Supplementary Figure 2 | Bright-field optical microscopy images of organic microspheres with different sizes.

Supplementary Figure 3 | Size distributions of spherical microcavities fabricated by the PS solution with different concentration.

III. Synthesis procedure and luminescence properties of the model compounds

Supplementary Figure 4 | The synthetic route of compound CNDPASDB

Supplementary Figure 5 | Molecular structures and spectral data of the selected laser dyes.

IV. Lasing measurements of isolated microsphere

Supplementary Figure 6 | Bright-field optical microscopy images of organic microspheres doped with different laser dyes.

Supplementary Figure 7 | Schematic illustration of the experimental setup for the optical characterization.

Supplementary Figure 8 | Microcavity effect of the organic microsphere.

V. Construction and lasing performances of the heterogeneously coupled WGM resonators

Supplementary Figure 9 | Schematic diagram of the fabrication process of the heterogeneously coupled microcavities.

Supplementary Figure 10 | Controllable fabrication of the heterogeneously coupled WGM microcavities.

Supplementary Figure 11 | Schematic diagram of the construction strategy of the desired gap distance for heterogeneously coupled WGM resonators.

Supplementary Figure 12 | Coupling effect of heterogeneously coupled WGM cavities.

Supplementary Figure 13 | Lasing characterization of the heterogeneously coupled WGM resonators with varying gap distances.

Supplementary Figure 14 | Evolution of the emission spectra with the increase of pump power for heterogeneously coupled WGM resonators and plots of PL peak intensities and full width at half-maximum (FWHM) versus the pump fluence.

Supplementary Figure 15 | Preparation of the heterogeneously coupled system composed of red and green emissive spherical microcavities

I. Characterization

Simulation. The electric field intensity distributions were obtained using the finite- difference time-domain (FDTD) method and the FDTD source code can be accessed from http://www.fdtdxx.com.

Morphological characterization. The morphology of the prepared microspheres was examined with a scanning electron microscope (SEM, Hitachi, S-4800).

Optical characterization. The fluorescence spectra were measured on a UV-visible spectrometer (Perkin-Elmer Lambda 35) and a fluorescence spectrometer (Hitachi F-7000), respectively. Bright-field optical images and fluorescence microscopy images were taken with an inverted fluorescence microscope (Nikon Ti-U) under the excitation of a mercury lamp (330-380 nm). A focused 400 nm pulse laser beam (200 fs, 1000 Hz) was generated from a regenerative amplifier (Spitfire, Spectra Physics, 800 nm, 200 fs, 1 kHz), which was in turn seeded by a mode-locked Ti: sapphire laser (Mai Tai, Spectra Physics, 800 nm, 120 fs, 80 MHz). The excitation laser was focused down to a 2 μm diameter spot through an objective lens (Olympus LMPLFLN, 50 ×, N.A. = 0.5). The power at the input was altered by a neutral density filter. The emissions from the individual microstructures were collected by the same objective in a back-scattering configuration. The spatially resolved spectra were recorded by a spectrometer after removing the excitation beam with a 430-nm long-pass filter.

II. Morphological characterization of spherical microcavities

The spherical microcavities were synthesized through a controlled emulsion-solvent-evaporation method. **Supplementary Fig. 1** displays the top- and side-view of the microsphere, indicating that the self-assembled microcavities have a spherical shape and perfectly smooth surface, which are favorable for the Whispering-gallery-mode (WGM) resonance. The diameter of self-assembled WGM resonators can be finely tuned from 3 to 20 μm through increasing the concentration of polystyrene (PS), which is critical for the construction of optimized heterogeneously coupled cavity system. Bright-field optical microscopy images of organic microspheres with different sizes are shown in **Supplementary Fig. 2**. The size distributions of spherical microcavities were provided in **Supplementary Fig. 3.** With smooth surface and controllable size, self-assembled microspheres are ideal candidates for constructing the well-designed heterogeneously coupled cavity system.

a b

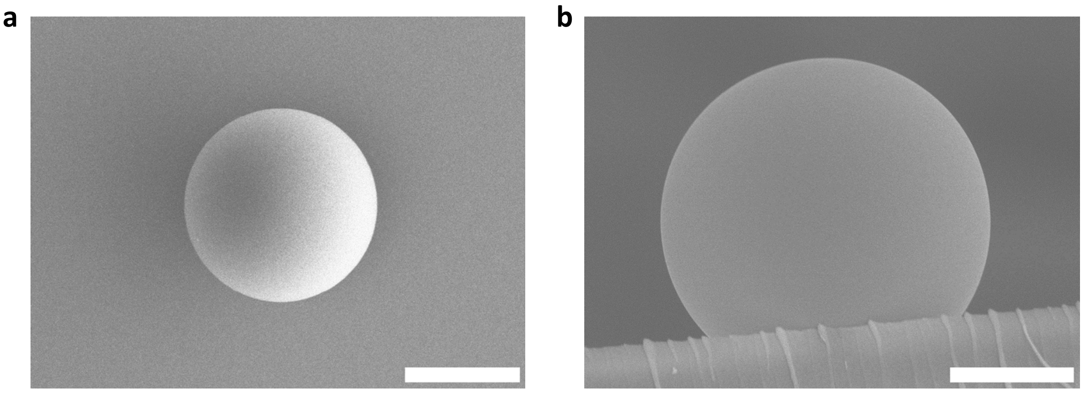

Supplementary Figure 1 | Scanning electron microscopy (SEM) images of the PS microspheres. **a**, top-view image, **b**, side-view image. Scale bars are 5 μm.

As shown in Supplementary Fig. 1a, the acquired structures have perfect circle boundary and ultra-smooth surface. The spherical structure is further confirmed by the side-view SEM image (Supplementary Fig. 1b). Such a microsphere is favorable for the whispering-gallery-mode (WGM) resonance.[1]

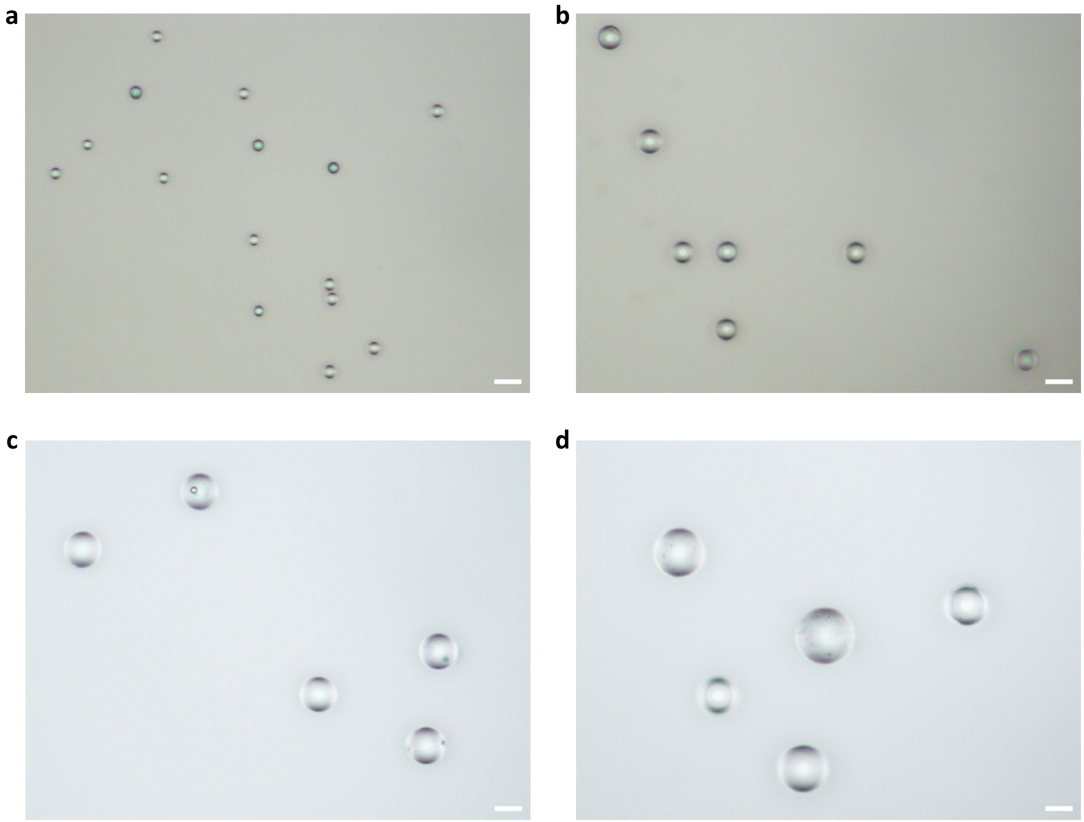

Supplementary Figure 2 | Bright-field optical microscopy images of organic microspheres with different sizes. **a**, 3 μm; **b**, 8 μm; **c**, 13 μm; **d**, 20 μm. All scale bars are 10 μm.

The diameter of the organic microspheres can be well tuned from 3 to 20 μm by changing the concentration of PS. When the concentration of PS was 10, 20, 30 and 50 mg mL^{-1}, the diameters of self-assembled organic microspheres were controlled to be ~3 μm (Supplementary Fig. 2a), 8 μm (Supplementary Fig. 2b), 13 μm (Supplementary Fig. 2c) and 20 μm (Supplementary Fig. 2d), respectively, which could be utilized to construct the optimized coupled structure.

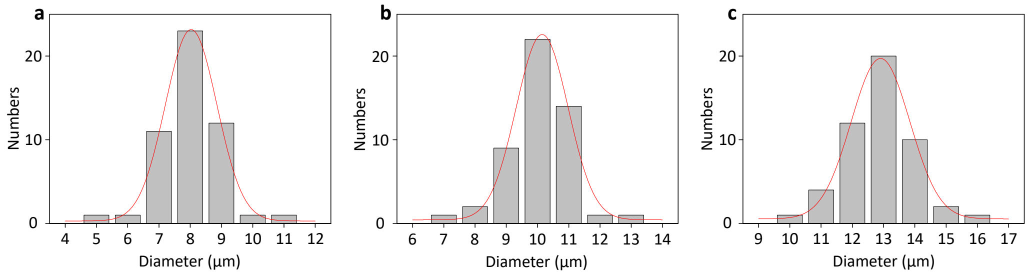

Supplementary Figure 3 | Size distributions of spherical microcavities fabricated by the PS solution with different concentration. **a**, 20 mg mL^{-1}, **b**, 25 mg mL^{-1}, **c**, 30 mg mL^{-1}.

To investigate the size distribution, spherical microcavities was fabricated by the PS solution with different concentration (20, 25 and 30 mg mL^{-1}) and the diameters of 150 spherical microcavities were measured. As shown in Supplementary Figure 3, when the concentration of PS was 20, 25 and 30 mg mL^{-1}, the mean diameter of the spherical microcavities were 8 μm (S.D. 0.825), 10 μm (S.D. 0.831) and 13 μm (S.D. 0.939), respectively. Such distribution can be fairly fitted by a Gaussian curve, indicating that the spherical microcavities is an ideal platform for constructing the heterogeneously coupled system.

III. Synthesis procedure and luminescence properties of the model compounds.

Due to the strong π-π interactions between the phenyl groups of PS and π-conjugated dye molecules, the microspheres can be doped with various conjugated dyes to provide optical gains at different wavebands. Accordingly, three π-conjugated luminescent dyes, C153, CNDPASDB, and DCM, with photoluminescence (PL) emission across the visible region, were selected as gain medium. The CNDPASDB were synthesized with Knoevenagel condensation reactions (**Supplementary Fig. 4**). The spectral data of the selected laser dyes are presented in **Supplementary Fig. 5**.

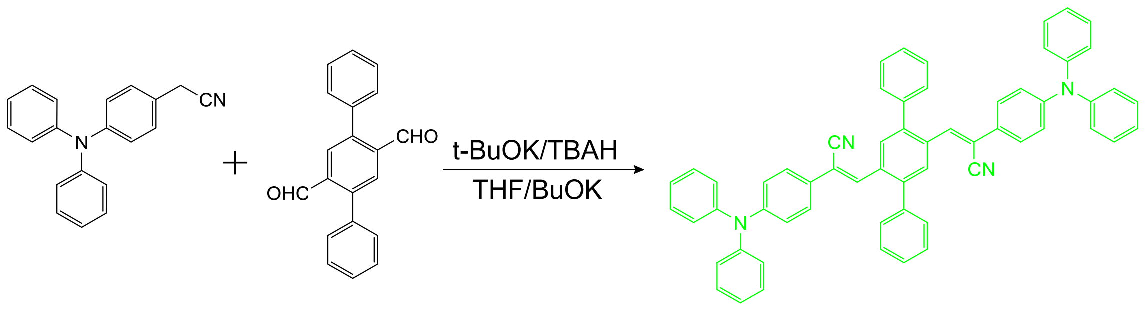

Supplementary Figure 4 | The synthetic route of compound CNDPASDB.

Step 1: Synthesis of [1,1';4',1'']terphenyl-2',5'-dicarbaldehyde

A mixture of 2,5-dibromobenzene-1,4-dicarbaldehyde (1.0 g), phenylboronic acid (1.1 g), $Pd(PPh_3)_4$ (0.2 g), toluene (12.5 mL) and 2 M Na_2CO_3 solution (2.5 mL) was refluxed at 85 °C for 36 hours under nitrogen, then poured into water and extracted using dichloromethane. The organic layer was washed with brine and water and dried over $MgSO_4$. The crude product was purified by flash column chromatography with dichloromethane as eluent. After recrystallized form chloroform, compound [1,1';4',1'']terphenyl-2',5'-dicarbaldehyde was obtained in 81% yield.

Step 2: Synthesis of 2-(cyanomethyl)-4-(diphenylamino)benzene[2]

2-(Cyanomethyl)-4-(diphenylamino)benzene was prepared from 4-(diphenylamino)benzaldehyde upon treatment with tosylmethylisocyanide (TosMIC) and t-BuOK in one single step.

Step 3: Synthesis of CNDPASDB[3]

2-(Cyanomethyl)-4-(diphenylamino)benzene (0.21 mmol) and [1,1';4',1'']terphenyl-2',5'-dicarbaldehyde (0.1 mmol) were dissolved in tert-butanol (1.2 mL) and THF (0.8 mL) under a nitrogen atmosphere. Potassium

tert-butoxide (0.02 mmol) and tetra-n-butylammonium hydroxide (0.02 mmol, 1 M solution in methanol) were added quickly; then, the mixture was stirred vigorously at 50 °C. After 20 min, the mixture was poured into acidified methanol. The crude product was precipitated from methanol and further purified by column chromatography under rigorous exclusion of light.

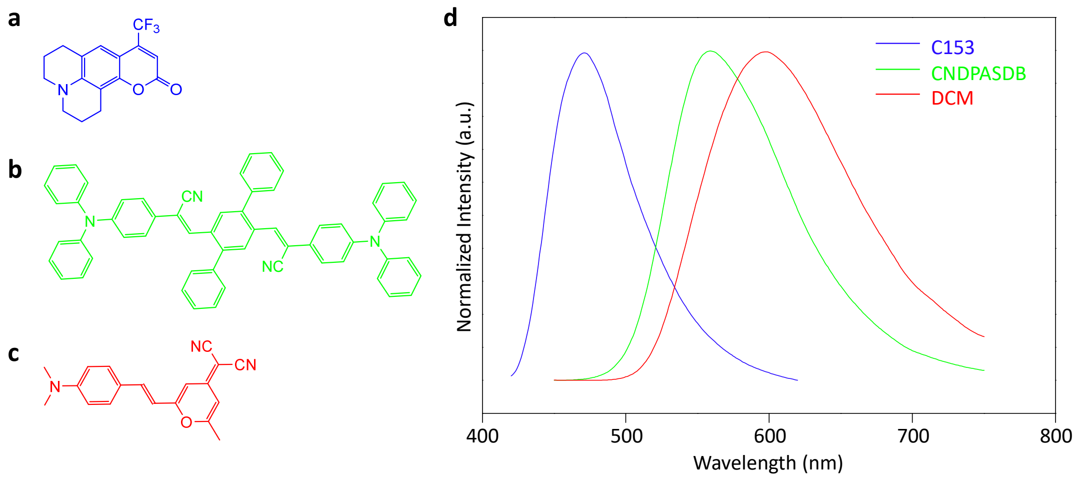

Supplementary Figure 5 | Molecular structures and spectral data of the three laser dyes. **a-c**, Molecular structures of C153 (**a**), CNDPASDB (**b**), DCM (**c**), respectively. **d**, Normalized PL spectra of the three laser dyes in PS. All concentrations of the three dyes are 0.01 mM.

Supplementary Fig. 4 presents the molecular structures and spectral data of the selected laser dyes. The PL spectra of the three laser dyes (C153, CNDPASDB and DCM) in PS span the RGB color, with the maximum emission at ~472 nm, ~560 nm and 600 nm, respectively, which offers us an opportunity to achieve microlasers in RGB wavebands.

IV. Lasing measurements of isolated microsphere.

The bright-field optical microscopy images of organic microspheres doped with different laser dyes in **Supplementary Fig. 6** demonstrated that the dopants introduced very little surface damage to the WGM resonators. **Supplementary Fig. 7** schematically shows the experimental setup for the optical characterization. The CNDPASDB-doped microspheres can be used to achieve multimode laser, while the diameter of microsphere and the mode space exhibit strong dependence, as shown in **Supplementary Fig. 8**. Such an inversely proportional relationship between the mode space and the diameter of the microsphere further verified the WGM resonance.

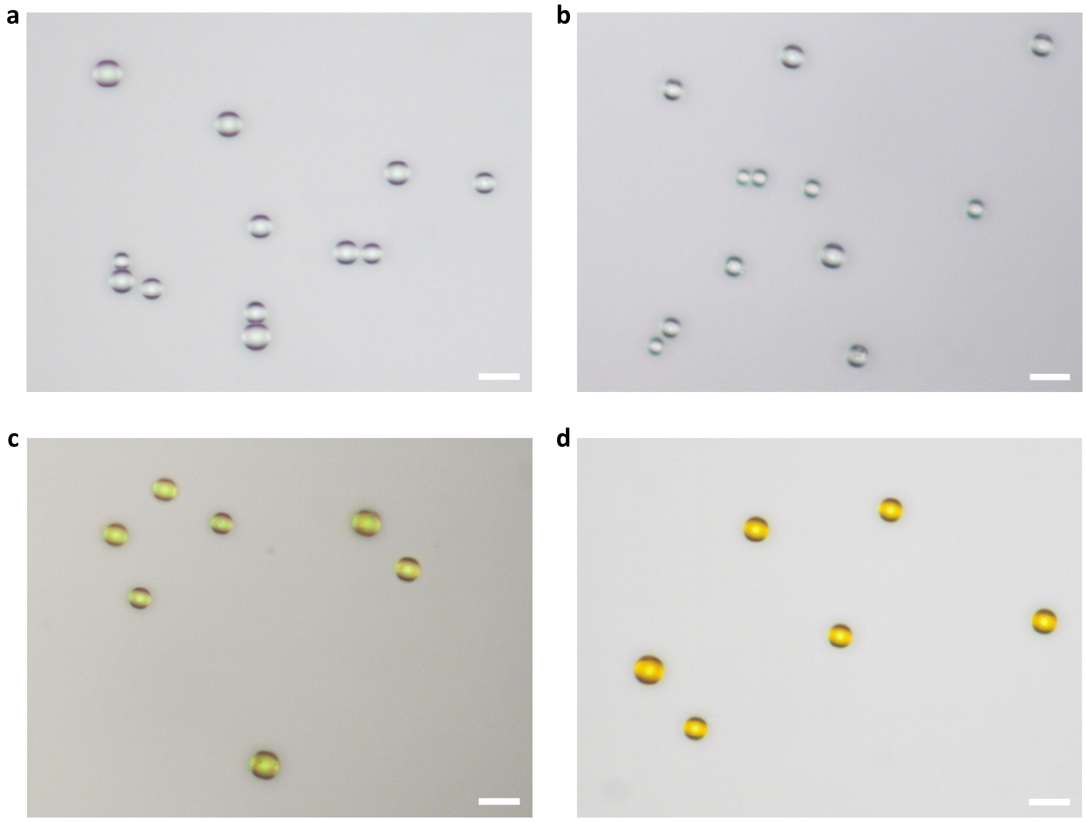

Supplementary Figure 6 | Bright-field optical microscopy images of organic microspheres doped with different laser dyes. **a**, microspheres without dopants, **b**, C153-doped microspheres, **c**, CNDPASDB-doped microspheres and **d**, DCM-doped microspheres. Scale bars are 5 μm.

Supplementary Fig. 5a shows the bright-field optical microscopy image of organic microspheres without dopants. Supplementary Fig. 5b-d show the bright-field optical microscopy images of organic microspheres doped with C153, CNDPASDB and DCM, respectively. The self-assembled microspheres doped with different laser dyes have a regular diameter and perfect circle boundary, which indicates that the dopants would not influence the sizes and boundaries of microcavities.

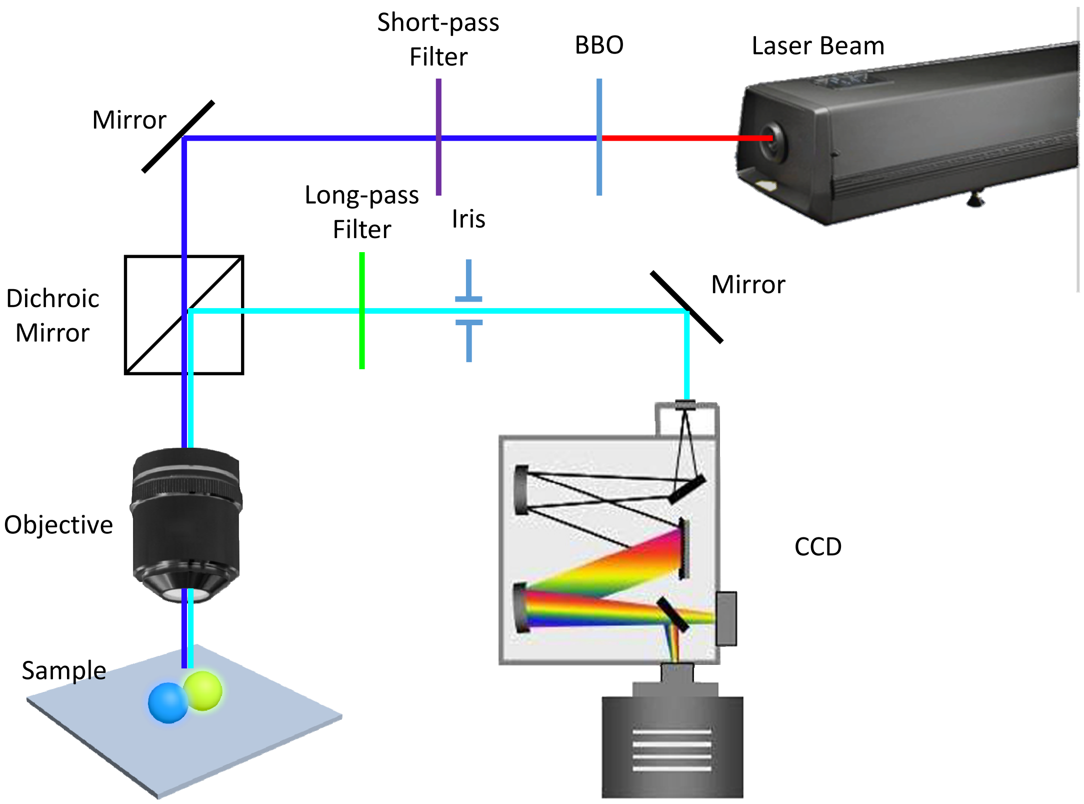

Supplementary Figure 7 | Schematic illustration of the experimental setup for the optical characterization.

A home-built microphotoluminescence system was used to exam the optical properties of the heterogeneously coupled WGM resonators. A focused 400 nm pulse laser beam, which was generated from the second harmonic of the fundamental output of a regenerative amplifier (Solstice, Spectra-Physics, 800 nm, 100 fs, 1000 Hz), was used to pump the heterogeneously coupled system. An objective lens (50×, numerical aperture 0.8) was used to focus the pump beam. The PL signal was collected with an objective (Nikon CFLU Plan, 20 ×, N.A. = 0.5). After passing through the corresponding filters (400-nm long-pass), the collected emissions were dispersed with a grating (1200 G/mm) and the recorded using a

thermal-electrically cooled CCD (Princeton Instruments, ProEm 1600B).

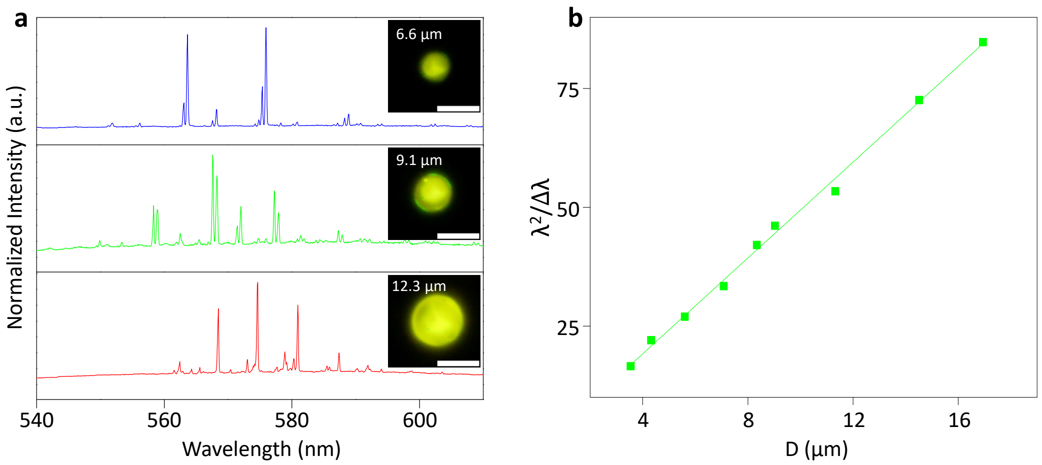

Supplementary Figure 8 | Microcavity effect of the organic microspheres. **a**, PL spectra of microspheres with different diameters and corresponding PL images. All scale bars are 10 µm. **b**, Relationship between $\lambda^2/\Delta\lambda$ and the diameter of the microsphere. The black line is fit to the function $\lambda^2/\Delta\lambda = n\pi D$.

The lasing spectra of microspheres with different diameters were measured, which presented an increasing number of lasing modes with the enlargement of the diameter of the microsphere. According to the WGM theory, the mode spacing, $\Delta\lambda$, and the cavity length, L, would satisfy the equation $\lambda^2/\Delta\lambda = n\pi D$, where λ is the wavelength of the guided light, $\Delta\lambda$ is the mode spacing, n is the group refractive index and D is the diameter of the cavity.[4] The plot of $\lambda^2/\Delta\lambda$ versus D demonstrated clearly a linear relationship, indicating that the lasing mechanism could be ascribed to WGM resonance.

V. Construction and lasing performances of the heterogeneously coupled WGM resonators

The controllable fabrication process of the heterogeneously coupled microcavity system is shown in **Supplementary Fig. 9** and **Supplementary Fig. 10**. The heterogeneously coupled microcavities with desired gap distances were constructed by alternately exerting an axial force to one of the microspheres in the micromanipulation process, as illustrated in **Supplementary Fig. 11**. The coupling effect of heterogeneously coupled WGM cavities was shown in **Supplementary Fig. 12**. The influence of the gap distance on mode modulation effect are presented experimentally in **Supplementary Fig. 13**. **Supplementary Fig. 14** presents the pump power-dependent lasing spectra of the heterogeneously coupled microspheres. The construction of the heterogeneously coupled system composed of red and green emissive microcavities was shown in **Supplementary Figure 15**.

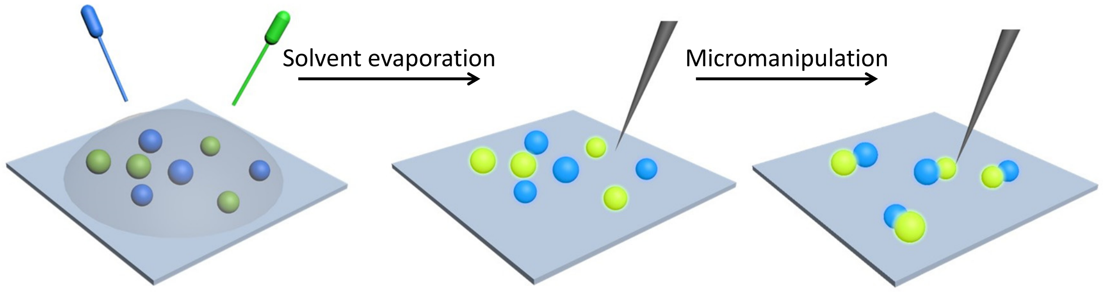

Supplementary Figure 9 | Schematic diagram of the fabrication process of the heterogeneously coupled microcavities.

The heterogeneously coupled microcavities system were prepared via a micromanipulation method. In a typical construction, the microspheres doped with C153 and microspheres doped with CNDPASDB were first transferred onto a glass substrate by dropping and evaporating the aqueous solutions on the substrate. The microspheres scattered on the substrate were then manipulated to construct the desired heterogeneously coupled WGM resonators by using a microprobe under an optical microscope equipped with super-long working distance objective.

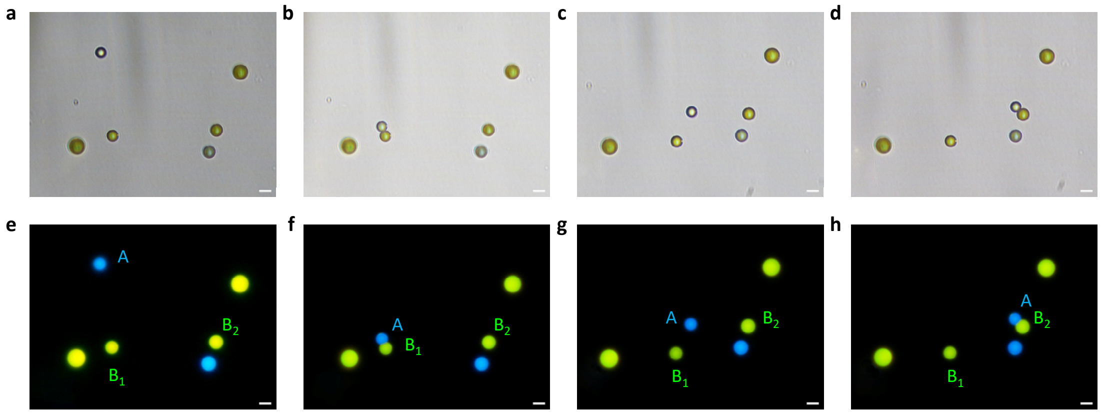

Supplementary Figure 10 | Controllable fabrication of the heterogeneously coupled WGM microcavities. **a-d**, Bright-field microscopy of the microspheres at different preparation stages. **e-h**, Corresponding PL images of the microspheres at different preparation stages. Scale bars are 5 μm.

The micromanipulation was carried out using a tungsten probe mounted on a precisely controlled three-dimensional moving stage under an optical microscope equipped with a super-long working distance objective. Bright-field microscopy and PL images show the fabrication process of the heterogeneously coupled WGM microcavities, with a certain microsphere, A, being controllably coupled to different microspheres, B_1 and B_2. This result indicates that heterogeneously coupled system composed of resonators with desired diameters can be constructed with such micromanipulation technique.

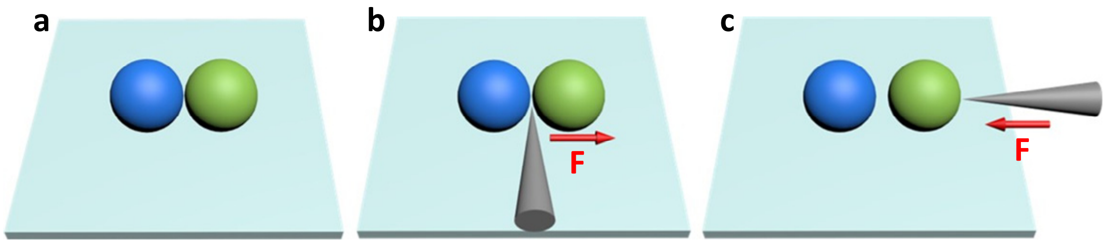

Supplementary Figure 11 | Schematic diagram of the construction strategy of the desired gap distance for heterogeneously coupled WGM resonators.

We constructed the heterogeneously coupled microspheres with desired gap distances by alternately exerting an axial force to one of the microspheres in the micromanipulation process, as illustrated in Supplementary Fig. 10. First, we push two heterogeneous microspheres together by using a microprobe to fabricate the heterogeneously coupled structures with no/little space between two component microspheres (Supplementary Fig. 10a). Next, one of the microspheres was pushed away from the axial direction by exerting a force to one of the microspheres (Supplementary Fig. 10b). Finally, the microsphere was move backwards by a force in the opposite direction, which would result in a gap with a desired distance (Supplementary Fig. 10c). This axial force technique enables us to control the gap distance in the range of effective coupling distances.

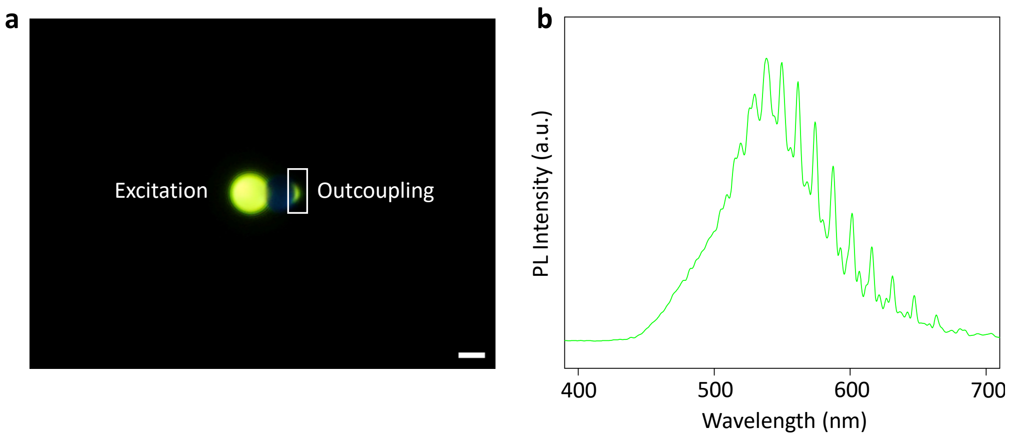

Supplementary Figure 12 | Coupling effect of heterogeneously coupled WGM cavities. **a**, PL image of the heterogeneously coupled WGM cavities locally excited at the CNDPASDB-doped microsphere. Scale bar is 5 μm. **b**, Spatially resolved PL spectra collected at rectangle a, as marked in (Outcoupling).

Supplementary Fig. 11 displays the PL photograph locally excited at the CNDPASDB-doped microsphere in heterogeneously coupled system. As shown in the PL image, the light emitted in the CNDPASDB-doped microsphere can be effectively coupled between them and outcoupled from the C153-doped microsphere. This is further proven by the spectral characterization in which the emission from CNDPASDB-doped microsphere is detected from the other unexcited microsphere.

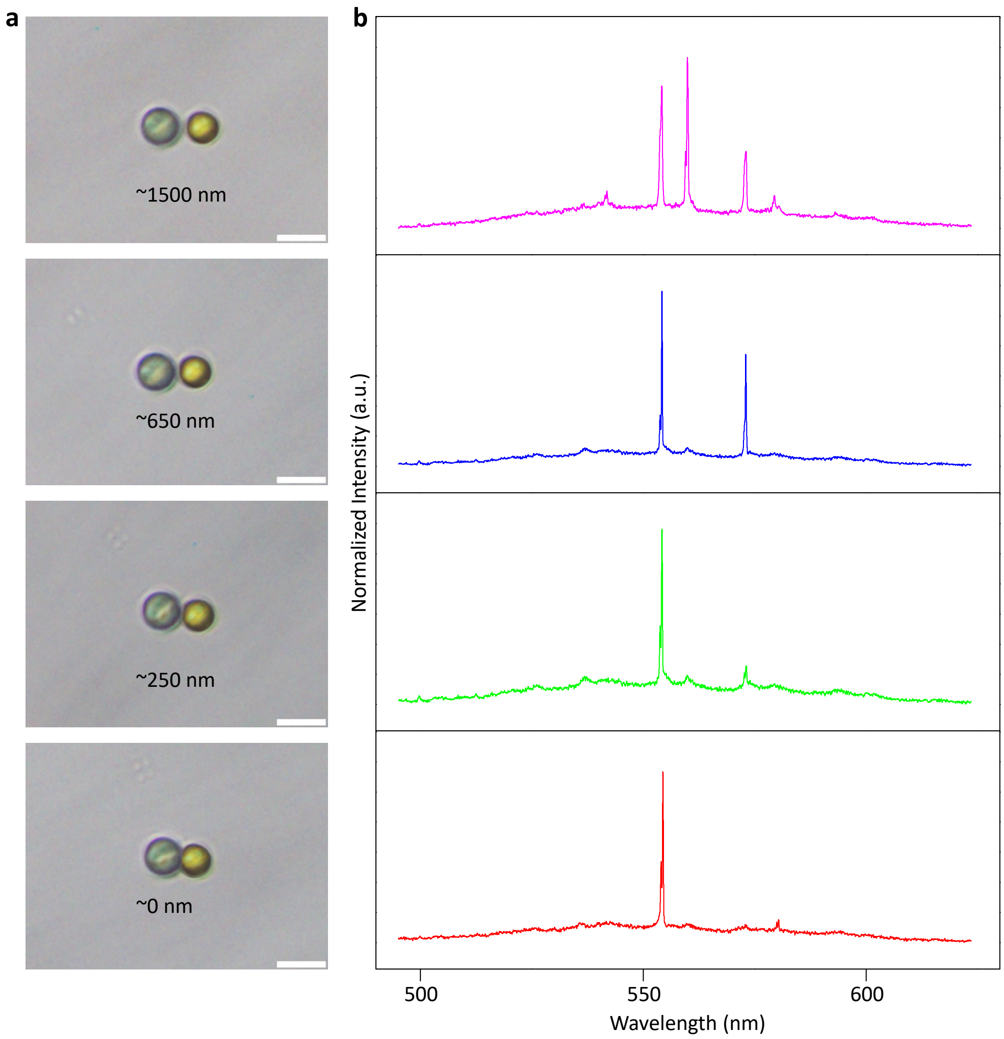

Supplementary Figure 13 | Lasing characterization of the heterogeneously coupled WGM resonators with varying gap distances. **a**, Optical images of the heterogeneously coupled microspheres with decreasing gap distance. Scale bars are 5 μm. **b**, Corresponding lasing spectra of the CNDPASDB-doped microsphere under different conditions.

The mode selection effect has a low requirement on the gap distance in coupled cavity system.[5] Here, we studied the evolution of the laser spectrum by gradually changing the gap distance of the heterogeneously coupled microspheres. A CNDPASDB-doped microsphere was selected as the lasing cavity, while a

C153-doped microsphere as the mode filter. As shown in Supplementary Fig. 12, the mode selection effect can be achieved in a range of gap distance ~0-~250 nm. When the gap width reaches ~600nm, more modes began to emerge because of the decrease of optical coupling between two microspheres. When the gap width reaches ~1200nm, there is no obvious mode selection effect anymore. This result indicates that single-mode microlaser can be steadily outputted when the gap distance between coupled microcavities varied from 0 to 250 nm, manifesting that the mode selection effect in heterogeneously coupled system has a low requirement on the gap distance.

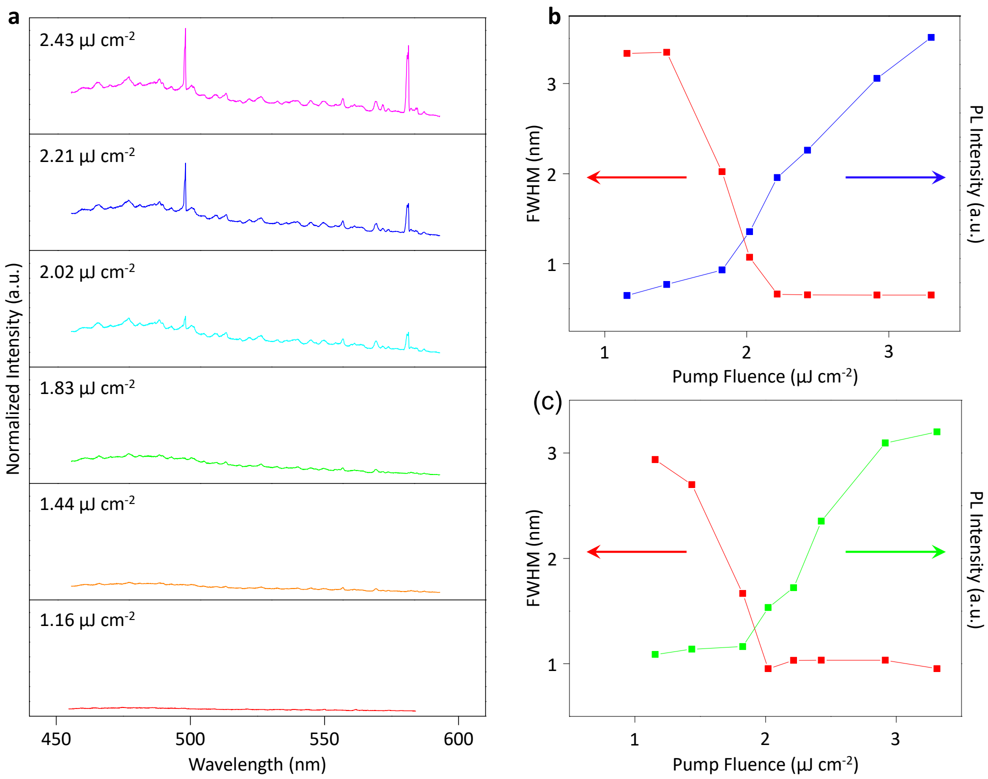

Supplementary Figure 14 | Evolution of the emission spectra with the increase of pump power for heterogeneously coupled WGM resonators and plots of PL peak intensities and full width at half-maximum (FWHM) versus the pump fluence. **a**, PL spectra of the coupled C153-doped and CNDPASDB-doped microspheres at different pump fluences. **b**, Plots of PL peak intensities (blue line) and FWHM (red line) of C153-doped resonator in coupled system at different pump fluences. **c**, Plots of PL peak intensities (green line) and FWHM (red line) of CNDPASDB-doped resonator in coupled system at different pump fluences.

As shown in Supplementary Fig. 13a, multicolor single-mode lasing actions were realized in the heterogeneously coupled WGM resonators. Supplementary Fig. 13b and Supplementary Fig. 13c depicted the PL intensity and the FWHM at 495nm and 572nm as a function of pump fluence, showing a threshold

characteristic at ~2μJ cm^{-1}. Above the onset power, the peak intensity increased rapidly with the pump fluence, and the FWHM dramatically decreased down to ~0.9 nm, which revealed the multicolor single-mode lasing behavior in heterogeneously coupled resonators.

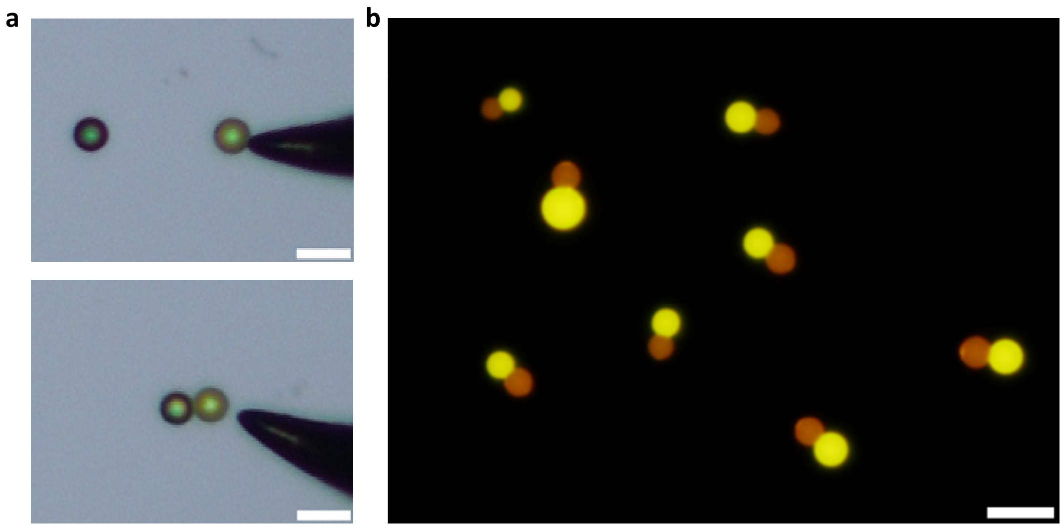

Supplementary Figure 15 | Preparation of the heterogeneously coupled system composed of red and green emissive spherical microcavities. **a,** Construction process of the heterogeneously coupled system composed of red and green emissive microcavities. **b,** PL image of the heterogeneously coupled microspheres under UV excitation. All scale bars are 10 μm

The construction of the heterogeneously coupled system composed of red and green emissive microcavities was shown in Supplementary Figure 15a. According to the manipulation strategy we mentioned in Supplementary Figure 11, heterogeneously coupled system constructed with red and green emissive resonators of different sizes were shown in Supplementary Figure 15b, indicating that the heterogeneously coupled system composed of resonators with desired diameters can be constructed with such micromanipulation technique

References

1. Wei, C., Gao, M., Hu, F., Yao, J. & Zhao, Y. S. Excimer Emission in Self-assembled organic spherical microstructures: an effective approach to wavelength switchable microlasers. **4,** *Adv. Opt. Mater.* 1009–1014 (2016).
2. He, F. et al. Diphenylamine-substituted cruciform oligo(phenylene vinylene)s: enhanced one- and two-photon excited fluorescence in the solid state. *Adv. Funct. Mater.* **17,** 1551–1557 (2007).
3. Li, Y. et al. Supramolecular network conducting the formation of uniaxially oriented molecular crystal of cyano substituted oligo(p-phenylene vinylene) and its amplified spontaneous emission (ASE) behavior. *Chem. Mater.* **20,** 7312–7318 (2008).
4. Samuel, I. D. W. & Turnbull, G. A. Organic semiconductor lasers. *Chem. Rev.* **107,** 1272–1295 (2007).
5. Zhang, C. et al. Dual-color single-mode lasing in axially coupled organic nanowire resonators. *Sci. Adv.* **3,** e1700225 (2017).
